# *IGHV1-69*-Encoded Antibodies Expressed in Chronic Lymphocytic Leukemia React with Malondialdehyde–Acetaldehyde Adduct, an Immunodominant Oxidation-Specific Epitope

**DOI:** 10.1371/journal.pone.0065203

**Published:** 2013-06-20

**Authors:** Xuchu Que, George F. Widhopf II, Shahzada Amir, Karsten Hartvigsen, Lotte F. Hansen, Douglas Woelkers, Sotirios Tsimikas, Christoph J. Binder, Thomas J. Kipps, Joseph L. Witztum

**Affiliations:** 1 Department of Medicine, University of California San Diego, La Jolla, California, United States of America; 2 Moores Cancer Center, University of California San Diego, La Jolla, California, United States of America; 3 Department of Laboratory Medicine, Medical University of Vienna, Vienna, Austria; 4 Department of Biomedical Sciences, University of Copenhagen, Copenhagen, Denmark; 5 Department of Reproductive Medicine, University of California San Diego, La Jolla, California, United States of America; 6 Center for Molecular Medicine (CeMM) of the Austrian Academy of Sciences, Vienna, Austria; Northwestern University Feinberg School of Medicine, United States of America

## Abstract

The immunoglobulins expressed by chronic lymphocytic leukemia (CLL) B cells are highly restricted, suggesting they are selected for binding either self or foreign antigen. Of the immunoglobulin heavy-chain variable (IGHV) genes expressed in CLL, *IGHV1-69* is the most common, and often is expressed with little or no somatic mutation, and restricted IGHD and IGHJ gene usage. We found that antibodies encoded by one particular IGHV1-69 subset, designated CLL69C, with the HCDR3 encoded by the *IGHD3-3* gene in reading frame 2 and *IGHJ6*, specifically bound to oxidation-specific epitopes (OSE), which are products of enhanced lipid peroxidation and a major target of innate natural antibodies. Specifically, CLL69C bound immunodominant OSE adducts termed MAA (malondialdehyde–acetaldehyde-adducts), which are found on apoptotic cells, inflammatory tissues, and atherosclerotic lesions. It also reacted specifically with MAA-specific peptide mimotopes. Light chain shuffling indicated that non-stochastically paired L chain of IGLV3-9 contributes to the antigen binding of CLL69C. A nearly identical CLL69C Ig heavy chain was identified from an MAA-enriched umbilical cord phage displayed Fab library, and a derived Fab with the same HCDR3 rearrangement displayed identical MAA-binding properties. These data support the concept that OSE (MAA-epitopes), which are ubiquitous products of inflammation, may play a role in clonal selection and expansion of CLL B cells.

## Introduction

Chronic lymphocytic leukemia (CLL) is a malignancy of monoclonal CD5^+^ B cells, which accumulate in the blood, marrow, and lymphoid tissues [1]. Previous studies found the immunoglobulin (Ig) repertoire expressed in this disease is highly restricted [2], [3], [4], [5], [6]. In general, CLL patients that express unmutated Ig heavy chain (IGHV) genes have a worse prognosis than those who express mutated IGHV genes [3], [7]. One particular IGHV gene, *IGHV1-69*, generally is expressed with little or no somatic mutation and is used by CLL cells of approximately 15% of all patients [3], [8], [9]. Moreover, the IGHV1-69-encoded Ig heavy chains (IgH) expressed in CLL commonly have stereotypic motifs in the third complementarity determining region (HCDR3), resulting from the rearrangement and restricted reading-frame-use of certain IGHD and IGHJ gene segments [4], [10], [11]. In prior studies, we identified four subsets of IGHV1-69-encoded IgH, expressed in CLL that each were preferentially paired with certain IgL, depending upon their respective HCDR3, [12] designated CLL69-A, -B, -C, and –D (also known as subsets 6, 3, 7H and 3, respectively) [9]. CLL69-A IgH encoded by IGHV1-69/IGHD3-16/IGHJ3 were co-expressed with light chains encoded by IGKV3-20 in all cases and CLL69C heavy chains encoded by IGHV1-69/IGHD3-3/IGHJ6 were paired with IgL encoded by IGLV3-9 in 77% of cases [12]. The prevalent use of unmutated IGHV1-69 and nonstochastic pairing with light chains suggests that self- and/or common-environmental antigen(s) plays a role in the development and/or progression of CLL [12], [13].

Chronic inflammatory diseases, including atherosclerosis, are characterized by an enhanced oxidative state and the accumulation of oxidized low-density lipoproteins (OxLDL), apoptotic cells and apoptotic cellular debris, which also bear many oxidatively-modified lipids and proteins. We have termed such oxidation-related neo-antigens as “oxidation-specific-epitopes” (OSE), and shown that OSE are not only greatly enriched in atherosclerotic tissue, but in inflammatory or infectious-disease states [14]. Remarkably, OSE are a major target of innate immunity in general and specifically are the target of ∼ 20–30% of all IgM natural antibodies (NAbs) in mice and in human newborn cord blood [15].

Among the most common end-products of lipid peroxidation is the generation of malondialdehyde (MDA), which is highly reactive and in turn can form simple and complex adducts with proteins. We have shown that such MDA adducts are highly immunogenic and prevalent targets of NAbs in both mice and humans [15]. Among these, there are particularly proinflammatory and immunodominant MDA type adducts with lysines of proteins, termed MAA (malondialdehyde–acetaldehyde adducts), of which the 4-methyl-1,4-dihydropyridine-3,5-dicarbonyl type adduct is a major product [16], [17], [18]. It has been reported that MAA adducts represent the main antigens produced when proteins are modified with concentrations of MDA exceeding 5 µmol/mL [17], [18]. We have recently shown that MAA epitopes are immunodominant targets of IgM NAbs in mice and human newborn plasma [15]. In turn, these NAbs immunostain MAA epitopes on apoptotic cells, as well as in atherosclerotic or other inflammatory tissues [15], [18].

Because oxidative processes are ubiquitous, we hypothesized that these OSE might serve as a self-antigen(s) that could stimulate B cells bearing appropriate Ig receptors, thereby enhancing their survival and increasing their risk for incurring transforming events [19]. We identify here that MAA-epitopes strongly reacted with the recombinant antibody (rAb) of the CLL69C subset.

## Materials and Methods

### Antigen Preparation and Modifications

The study was reviewed and approved by the Human Research Committee of the University of California San Diego that conforms to the Declaration of Helsinki, and blood and/or tissue samples were collected with written informed consent from the donors (UCSD Human Research Protection Program #071402). For the studies described in this proposal, blood and/or tissue samples from human subjects >18 years of age who sign a consent form prior to undergoing a catherization and angioplasty for therapeutic correction of blockage of the blood vessel. Subjects recruited at UCSD will provide blood samples (∼50 ml of blood) and/or any tissue that is retrieved during the procedure deposited on a distal protection device used distal to the site of repair to prevent dislodged tissue from blocking the artery distal to the site of operation by the cardiologist. Subjects who agree to donate a blood sample or tissue during the therapeutic procedure will be asked to sign an IRB approved informed consent form giving permission for the studies as noted above. Less than 50 ml of blood will be collected. Subjects who agree to donate blood/tissue will be asked to sign an IRB approved consent form giving permission for blood/tissue collection. Human LDL was freshly isolated from plasma of healthy donors after an overnight fast by sequential ultra-centrifugation**,** and modified with MAA, MDA or CuSO4 to generate MAA-LDL, MDA-LDL or copper-oxidized LDL (Cu-OxLDL) respectively, as previously described [15]. Phosphocholine-BSA (PC-BSA) was from Biosearch Technologies.

### Peptide Mimotopes

We recently reported in detail the generation of small molecule peptide mimotopes of MAA [20]. In brief: To generate peptide mimotopes of MAA-epitopes present on MDA-LDL, peptide libraries purchased from New England Biolabs (NEB) (Beverly, MA, USA) were screened by biopanning for phages reactive with LRO4 [20]. LRO4 is a natural Ab that was cloned in our laboratory from cholesterol-fed *Ldlr^−/−^* mice and shown to bind MAA-modified BSA or MAA-LDL. Peptide mimotopes of MAA were synthesized by Peptide 2.0 Inc. (Chantilly, VA, USA). The purity of the all peptides was between 89–95% as assessed by high performance liquid chromatography and mass spectral analysis.

### Recombinant CLL69 Ab Production

The recombinant CLL69 Ab was produced in exponentially growing 293T human embryonic kidney cells that were co-transfected with equimolar amounts of IgH and IgL plasmid DNA expression vectors by calcium-phosphate precipitation as described [21], [22]. Purified recombinant IgG concentrations were determined by ELISA using human IgG1 as a standard.

### Chemiluminescent ELISA for Binding to OSE and Mimotopes by CLL69 rAb and Fab

Antibody (Ab) binding assays were performed using chemiluminescent technology as described [23] with modifications. Round-bottomed MicroFluor 96-well plates (DYNEX Technologies, Chantilly, VA) were coated with various antigens at 5 µg/mL (50 µl per well) in PBS overnight at 4°C. Synthetic peptides were directly coated at 10 µg/mL (P1) or 5 µg/mL (P2) in 0.1 M NaHCO_3_ buffer (pH 8.6), unless indicated differently. Biotinylated peptides were immobilized at indicated concentrations in 0.1 M NaHCO_3_ buffer (pH 8.6) on wells pre-coated with 10 µg/mL neutravidin (Pierce, Rockford, IL, USA). After the plates were washed and blocked with 1% BSA in PBS for 30 min, 25 µL of primary Abs diluted with 1% BSA-PBS were added to the wells, and incubated for 90 min at room temperature. Bound Abs were detected with isotype-specific goat anti-human IgG1 alkaline phosphatase conjugate (Southern Biotech), or alkaline phosphatase conjugated anti-HA mAb (SIGMA) for Fab, in Tris buffered saline (TBS) buffer containing 1% BSA, followed by a rinse with water and the addition of 25 µL of 50% LumiPhos 530 (Lumigen, Southfield, MI) as luminescent substrate. The quantitative readout of these assays is light emission, measured as relative light units (RLU) over 100 ms (RLU/100ms) using a Dynex Luminometer (DYNEX Technologies). All determinations were done in triplicate. The specificity of rAb binding was determined by competition chemiluminescent ELISA as described previously [23], and data expressed as B/B_0_, where B represents binding in the presence and B_0_ in the absence of competitor.

### CLL69 Homologous Gene Isolation and Sequence Analysis

An umbilical cord (UC) Fab phage display library was constructed as described by Barbas *et al.* [24] and will be reported on in detail elsewhere. For studies described here, the phage libraries were screened for binding to MAA-BSA and MDA-LDL, using the chemiluminescence immunoassay. Target distinct IGHV1-69 genes were amplified from the selected UC phage library using a CDR2-specific sense primer corresponding to the sequence encoding amino acid positions 50–58 of the IGHV1-69 allelic subsets (5′-AGGGATCATCCCTATCTTTGGTAC-3′) and an antisense primer for germline IGHJ6 consensus sequence (5′-GRGGAGACGGTGACCAGGGT-3′). The HCDR3 and flanking regions of IGHV1-69 genes were amplified using 5 µl of phage DNA template (10e10 phage) from each phage library panned for 4^th^-round of MAA-BSA. PCR products were analyzed on 2% agarose gel and subcloned into a TA cloning vector pCR2.1 (Invitrogen) for sequence determination. To isolate an IGHV1-69 homologous Fab clone, the UC Fab phage display library was also screened with a murine anti-idiotypic mAb G6 [25], which reacts only with IgH encoded by unmutated IGHV1-69 genes [10]. The reactive phage particles were selected by panning on ELISA plates coated with mAb G6 at 1 µg/well. After three rounds of panning, the phage vector was converted to the plasmid producing soluble Fab. Nucleotide and amino acid sequences of the IGHV from all clones were compared to those contained in public databases by using Ig-BLAST and IMGT/V-QUEST.

### Expression and Purification of Soluble Fab Ab

Plasmids were purified from selected Fab phage clones and transformed into *E. coli* BL21 (DE3) for production of soluble Fab. Purification of His_6_- and HA-tagged Fab Abs was carried using Ni-NTA Agarose (Qiagen) and anti-HA Agarose (Sigma) according to the manufacture’s protocols. Briefly, Fab Ab expression was induced with 1 mM isopropyl-D-thiogalactopyranoside in *E. coli* BL21 (DE3) that had been grown to mid-log phase in SuperBroth media. Following induction, bacteria were grown for 16–20 h at 30°C, harvested and resuspended in lysis buffer (1% of culture volume; 500 mM NaCl, 50 mM NaP*i* buffer pH 7.5, 0.05% Tween-20, 10 mM imidazole) containing protease inhibitors and lysozyme (200 µg/mL) for 20 min on ice and sonicated for 6×10s. The lysate was clarified by centrifugation (20,000×*g*, 30 min, 4°C), and the supernatant was applied to Ni^2+^-NTA agarose beads (Qiagen), from which His6-tagged Fab constructs were eluted using lysis buffer supplemented with 250 mM imidazole. Fractions containing Fab constructs were pooled and dialyzed against PBS on concentrator column and next purified by application to anti-HA filtration chromatography in TBS buffer. After wash with PBS, the Fab Ab were eluted with 0.1 M glycine (pH 3.0). Elutes were neutralized by 2 M Tris-HCl, pH 9.0 in collected tubes. Purified Fab constructs were concentrated in PBS buffer.

### Flow Cytometry and Immunofluorescence Microscopy Analysis of Binding to Apoptotic Cells

CLL69 Abs and umbilical Fabs were analyzed for binding to apoptotic cells by flow cytometry analysis as described [15]. Thymocytes harvested from C57BL/6 mice were cultured in cell culture medium and induced to undergo apoptosis by 10 ng/ml PMA (Sigma-Aldrich) for 16 hours as described previously [26]. Apoptosis of Jurkat cells were prepared by exposure to UV irradiation at 20 mJ/cm^2^, and further cultured for 16 hours before use. CLL69 Abs or control Ab (1 µg/ml) diluted in 1% BSA-PBS were incubated with apoptotic thymocytes or Jurkat cells for 1 hr at 4°C, followed by incubation with FITC-labeled goat anti-human IgG1 (Southern Biotech) or FITC-labeled anti-HA mAb in 1% BSA-PBS for 30 minutes at 4°C. Apoptotic cells were double-stained with Annexin V - Phycoerythrin (Annexin V - PE) and 7-amino-actinomycin (7-AAD) (BD Biosciences) for 15 minutes and immediately analyzed by flow cytometry using a FACSCanto (BD Biosciences).

For immunofluorescence microscopy studies, the cells were incubated with CLL69 Abs, Fab or secondary Ab controls (as above) containing 1 µg/mL of Hoechst dye (Sigma-Aldrich). The cells were fixed with 3.7% paraformaldehyde for 20 minutes, washed, and re-suspended in PBS with 1%BSA. The cells were spun down on glass slides using cytospin (Thermo Shandon). Images were captured using a DeltaVision deconvolution microscopic system operated by SoftWorx software (Applied Precision) as described previously [15].

### Macrophage Binding Assay

Binding of biotinylated MAA-LDL to J774 macrophages plated in microtiter wells was assessed by a chemiluminescent binding assay as described previously [23]. Biotinylated MAA-LDL (5 µg/ml) was incubated in the absence or presence of CLL69 Ab or Fab at various dilutions overnight at 4°C. The supernatants were then added to macrophages and the binding of biotinylated MAA-LDL detected by AP-labeled NeutrAvidin and chemiluminescent ELISA.

### Immunohistochemistry

Immunostaining of formal sucrose-fixed, paraffin-embedded sections of aortas of atherosclerotic Watanabe heritable hyperlipidemic (WHHL) rabbits and human carotid atherosclerotic endarterectomy lesions was performed as described previously [27]. Sections of human or rabbit lesions were blocked with PBS containing 5% horse serum and 2% Fc blocker and stained with diluted CLL69 rAbs (1∶200), followed by addition of a biotinylated goat anti-human IgG1 (Southern Biotech) or anti-HA biotin mAb conjugate to detect the bound CLL69 Ab or Fab in the lesions. A Vectastain ABC- alkaline phosphatase kit and a Vector Red alkaline phosphatase chromogenic substrate (Vector Labs, Burlingame, CA, USA) were used to visualize Ab staining. Sections were counterstained with Weigert’s Iron Hematoxylin (Richard-Allan Scientific, Kalamazoo, MI, USA). Immunostaining of adjacent sections in the absence of primary Abs or VH3-21 rAb were used as negative controls. Endogenous tissue AP activity was blocked by 15 minutes of incubation with 5 mM levamisole (Sigma Chemical Co.).

## Results

### Oxidation-specific Epitopes are Dominant Targets of IGHV1-69-encoded Antibodies Expressed by CLL B Cells

**Figure 1:**
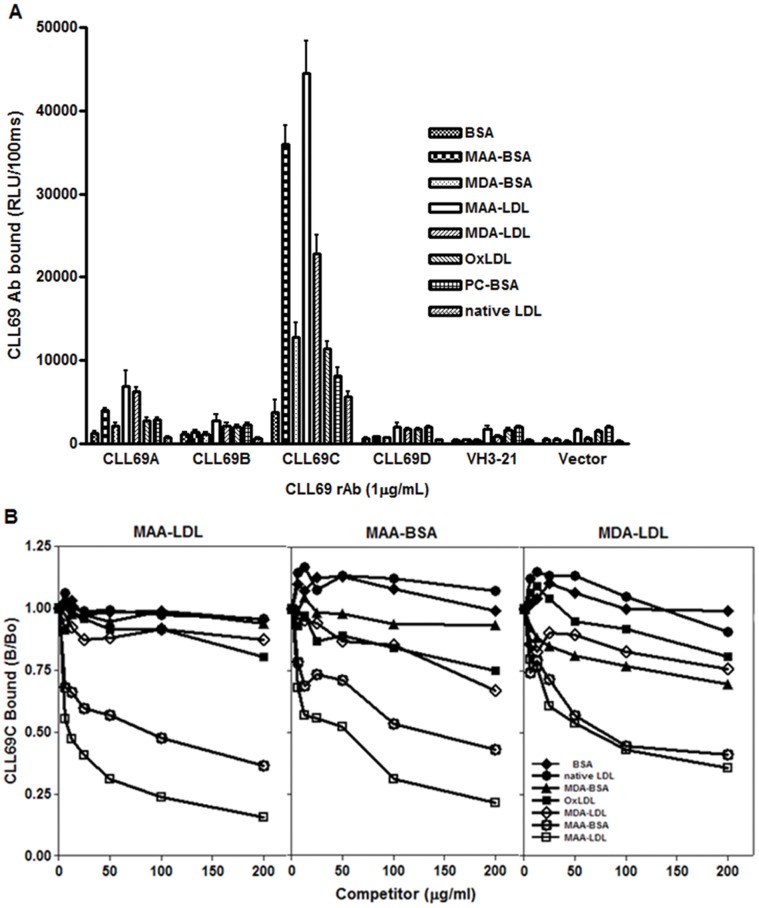
Binding pattern of CLL69 rAbs to OSE. (**A**) Chemiluminescent ELISA of CLL69 rAbs (1 µg/mL) binding to plated BSA, MAA- and MDA-modified BSA, MAA- or MDA-modified LDL, copper oxidized LDL (OxLDL), phosphocholine-modified BSA (PC-BSA), or native LDL (LDL), each at 5 µg/mL, for 1.5 hour at RT. Binding was determined as in methods, expressed as relative light units (RLU) per 100 milliseconds (ms) and represent at least three independent experiments in triplicate determinations (mean ± SD). (**B**) Competition immunoassay for the specificity of CLL69C rAb binding to MAA- epitopes. A fixed and limiting dilution of CLL69C rAb was incubated in the absence and presence of increasing amounts of indicated competitors and the extent of binding to plated MAA-LDL (left panel), MAA-BSA (middle panel) or MDA-LDL (right panel) was determined. Data are expressed as a ratio of binding in the presence of competitor (B) divided by absence of competitor (B_0_). Data are representative of three independent experiments, each determined in triplicate.

We examined recombinant antibodies from each of four previously identified subsets of IGHV1-69-encoded Ig heavy chains, designated as CLL69A, -B, -C, and –D, and tested them for binding to OxLDL and antigens modified with simple or complex MDA type epitopes. For this, we prepared simple, non-fluorescent MDA adducts (MDA-LDL and MDA-BSA) as well as complex, highly fluorescent MDA adducts, termed MAA (malondialdehyde-acetaldehyde adducts, MAA-LDL and MAA-BSA). Note that the MDA-modified preparations still contain a small number of fluorescent MAA adducts, and in turn, the MAA-modified preparations contain small numbers of simple MDA epitopes. Fig. 1A shows results from a direct Ab-binding assay of the four recombinant IGHV1-69-encoded rAbs, as well as control Abs, to the various antigens. Each of the four IGHV69-encoded rAbs bound predominantly to the highly fluorescent MAA epitopes. Although each of the four IGHV1-69 rAbs bound to MAA and MDA epitopes, CLL69C rAb had by far the greatest binding activity, for example, binding to MAA-epitopes (MAA-LDL and MAA-BSA) eight-fold higher than that of any of the other CLL69 subsets.

Among the various antigens tested, there was a clear preference for binding to the more complex MAA epitopes. Specifically, CLL69C rAb bound avidly to MAA-LDL and MAA-BSA, but also had lower binding to MDA-LDL and MDA-BSA, and to even a lesser degree to Cu-OxLDL, but did not bind to non-oxidized (native) LDL or BSA (Fig. 1A). CLL69A rAb, showed the next highest binding reactivity, although at much lower levels, having similar binding reactivity for MAA-LDL, MAA-BSA, or MDA-LDL, but not native LDL or BSA. CLL69B and CLL69D rAbs had minimal binding activity for each of a variety of oxidative epitopes on modified LDL or BSA. Both CLL69A and CLL69C exhibited dose-dependent binding to MAA-LDL (Figure S1, left panel) and at lower levels to MDA-LDL (Figure S1, right panel). In all assays, control rAbs with Ig heavy chains encoded by IGHV3-21, or samples without rAb, did not bind to any of the antigens.

The specificity of the binding of CLL69C rAb for MDA/MAA epitopes was verified in competition immunoassays in which binding to plated antigen was assessed in the presence of increasing concentration of soluble competitor antigen (Fig. 1B). The binding of CLL69C rAb to MAA-LDL (left panel) or to MAA-BSA (middle panel) was specifically competed only by MAA-epitopes (MAA-LDL or MAA-BSA), but not MDA-epitopes (MDA-LDL or MDA-BSA) or OxLDL, which is more enriched with oxidized phospholipid epitopes. Additionally, the binding of CLL69C rAb to MDA-LDL (right panel), which contains a low content of MAA epitopes, was competed mainly by antigens enriched in MAA epitopes (MAA-LDL or MAA-BSA). (It should be noted that the Y-axis in these competition data represent the percent competition of the competing antigen to inhibit binding of CLL69C to the indicated antigen. The CLL69C rAb had absolute binding activity for MDA-LDL that was much less than that for MAA-LDL, as shown in Fig. 1A). Collectively, these data demonstrate that CLL69C rAb binds to MAA epitopes, which are greatly enriched in MAA-modified proteins, and present to only a minor degree in MDA-modified preparations.

### The Light Chain of CLL69C Recombinant Antibody Contributes to MAA-adduct Binding

**Figure 2:**
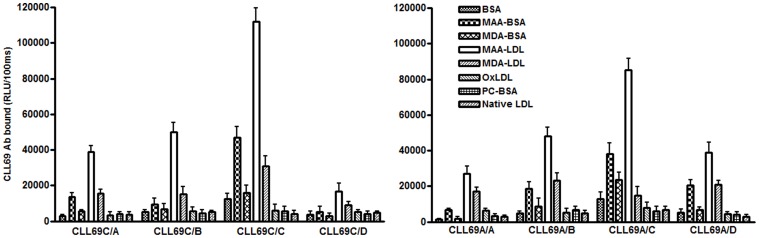
Binding pattern of CLL69C and CLL69A Ig heavy chains paired with non-native light chains to OSE. CLL69C or CLL69A rAb Ig heavy chains were paired with Ig light chains derived from other CLL69 subsets. Chemiluminescent immunoassays were used to determine the binding of each of the CLL69 rAbs (2 µg/ml) to the indicated plated antigens (5 µg/mL) for 1.5 hour at RT. Data are expressed as RLU/100 msec and are from three independent experiments (mean ± SD), with each value determined in triplicate.

IGHV1-69 subgroups contain HCDR3 with strikingly homologous amino acid sequences and, in many cases, these HCDR3 are associated with nearly identical Ig light chains, thereby forming BCRs that are identical or nearly-identical in unrelated patients. This suggests recognition of common epitopes in this subset. To examine the contribution of Ig light chains to the binding activity of CLL69C-encoded rAb for MAA epitopes, we assessed the relative binding activity for MAA-epitopes of rAb comprised of Ig heavy chains of CLL69C paired with non-native pairs of Ig light chains from CLL69A, B, or D. The results revealed that the recombinant CLL69C Ig heavy chain paired with its native IGLV3-9-encoded Ig light chain bound MAA-epitopes more effectively than the CLL69C IgH paired with non-native Ig light chains of CLL69A, B, or D (Fig. 2, left panel). However, CLL69A Ig heavy chains paired with the CLL69C IgL chain (IGLV3-9), designated CLL69A/C, exhibited three- to five-fold higher binding to the MAA-epitopes over the parental CLL69A/A recombinant Ab (Fig. 2, right panel). These observations suggest that the Ig light chain of CLL69C also contributes to the binding activity of rAb for MAA-epitopes.

### MAA-mimotopes are Recognized by CLL69 Abs

**Figure 3:**
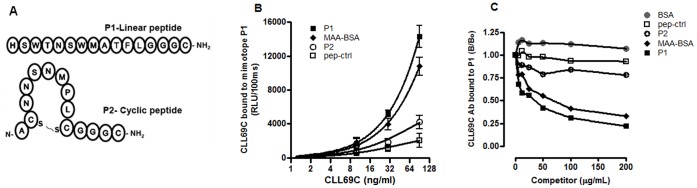
MAA-peptide mimotopes are recognized by CLL69C rAb. (**A**) Amino acid compositions of MAA-mimotopes P1 and P2 as recently reported [20]. (**B**) Chemiluminescent ELISA for the binding of increasing concentrations CLL69C rAb to peptide mimotopes P1 and P2, MAA-BSA, or an irrelevant control peptide (pep-ctrl). Data are expressed as RLU/100 msec, and are from three independent experiments (mean ± SD), each value determined in triplicate. (**C**) Competition immunoassay demonstrates specificity of the CLL69C rAb to mimotope peptide P1. A fixed and limiting concentration of CLL69C Ab was incubated in the absence or presence of increasing concentrations of indicated competitors, and extent of binding to P1-coated plates was determined and expressed as B/B0 from triplicate determinations (mean ± SD) as explained in legend to Fig. 1B.

We recently defined two short-chain peptide mimotopes for MAA-epitopes (P1 and P2) that were identified from phage peptide libraries, one consisting of a 12-mer linear peptide (P1- HSWTNSWMATFL), and the other being a cysteine-constrained heptamer cyclic peptide (P2-ACNNSNMPLC) (Fig. 3A). Each of these mimotopes were selected because of their ability to bind LR04, an MAA-specific mAb cloned in our lab, as recently reported [20]. We therefore tested whether the CLL69C rAb was also able to recognize these mimotopes. As shown in Fig. 3B, CLL69C rAb bound readily to P1 in a dose dependent manner, and to a more limited extent to P2, but not to a control scrambled peptide. As these peptides are small, and binding to microtiter wells might distort their conformation, they were biotinylated and bound to avidin-coated wells. Competition immunoassays demonstrated that binding of CLL69C rAb to P1 was effectively competed by increasing concentrations of soluble MAA-BSA and P1 peptide (Fig. 3C), but not by unmodified BSA or control peptide, confirming the high specificity of CLL69C rAb for the P1 MAA-mimotope.

### Molecular Analysis of IGHV1-69 Homologs from OSE Selected Umbilical Cord Fab Phage Library

IgM in newborn cord-blood are thought to best represent innate NAbs in humans. We have shown that OSE epitopes are a major target of the IgM in newborn cord blood, and that in particular, MAA-epitopes might account for up to 15% of all such IgM [15]. Therefore, we generated a human Fab phage display library using lymphocytes isolated from newborn cord-blood that would be enriched for innate NAbs binding to OSE. To investigate if the CLL69 subset gene homolog could be enriched from this library, we panned the umbilical cord Fab phage display library for 4-rounds with OSE (MAA-BSA and MDA-LDL), perhaps mimicking the natural selection of CLL69 BCR by neo-self-antigen epitopes. To isolate IGHV1-69-encoded heavy chains, the phages from the 4^th^-round MAA-BSA selected phage library were pooled and PCR amplified, using an oligonucleotide 5′-primer specific for the CDR2 of IGHV1-69 and a consensus 3′-primer of IGHJ6. Six unique clones were isolated and sequenced (Table 1), and two of them, UCL2 and UCL3, had high homology to CLL69C and CLL69B, respectively (Table 1). The other four clones were less well matched to any of the IGHV1-69-encoded Ig heavy chains that belong to subsets CLL69 A-D. UCL2 was 98% homologous to the germline IGVH1-69 gene, and best aligned to the stereotypic CLL69C HCDR3 sequence. The HCDR3 of UCL2 was encoded by the IGHV1-69, IGHD3-3, and IGHJ6 genes, with N insertions at both IGHV-IGHD and IGHD-IGHJ junctions, but is two amino acids shorter at the N1 insertion compared to CLL69C (Table 1). The UCL3 heavy chain was most homologous to the CLL69B HCDR3 sequence, as UCL3 and CLL69B both have HCDR3 encoded by IGHD2-2 and IGHJ6 and the amino acid motif DIVVVPAA encoded by the third reading frame of IGHD2-2 (Table 1). Thus, the unmutated prototypic CLL69 ancestors selected from the primary BCR repertoire of umbilical cord Fab library were similar to those of CLL69C expressed by leukemia B cells.

**Table 1:** The HCDR3 sequences of CLL69 homologs isolated from the MAA-BSA panned umbilical cord Fab phage display library.

Clone No.	IgHV	N1	D Segment	N2	JH	HCDR3 Length
	**IgHV1-69**		D1-20*01		JH6*01	
**UCL1**	TGTGCGAGA	CGTTTACGAC	ATAACTGGAACG	………	ACTACTACTACGGTATGGACGTCTGG	
	C A R	R L R	H N W N		D Y Y Y G M D V W	17
	**IgHV1-69**		D3-3*01		JH6*02	
**UCL2**	TGTGCGAGA	GGGG	ATTACGATTTTTGGAGTGGTT	CCC	ACTACTACTACTACGGTATGGACGTCTGG	20
	C A R	G	D Y D F W S G	S	H Y Y Y Y G M D V W	
	IgHV1-69*01		D3-3*01		JH6*02	
**CLL69C**	TGTGCGAGAG	CTGGAGGC	TACGATTTTTGGAGTGGTTATTATA	GTA	ACTACTACTACTACGGTATGGACGTCTGG	23
	C A R	A G G	Y D F W S G Y Y	S	N Y Y Y Y G M D V W	
	**IgHV1-69**		D2-2*02		JH6*01	
**UCL3**	TGTGCGAGAGA	GAAGG	AGGATATTGTAGTAGTACCAGCTGCCGTACC	A	TACTACTACTACGGTATGGACGTCTGG	23
	C A R	E K	E D I V V V P A A V P		Y Y Y Y G M D V W	
	IgHV1-69*01		D2-2*01		JH6*01	
**CLL69B**	TGTGCGAGAGA	CGTGCC	GGATATTGTAGTAGTACCAGCTGCTAT	T	TACTACTACTACGGTATGGACGTCTGG	22
	C A R	<1?tf="TT825c8005"?>D V P	D I V V V P A A I		Y Y Y Y G M D V W	
	**IgHV1-69**		D2-15*01		JH6*02	
**UCL4**	TGTGCGAGA	GTAG	ATTGTAGTGGTGGTAGCTGCTACTC	………	CTACTACTACTACGGTATGGACGTCTGG	20
	C A R	V	D C S G G S C Y S		Y Y Y Y G M D V W	
	**IgHV1-69**		D2-15*01		JH6*02	
**UCL5**	TGTGCGAG	CCCT	TATTGTAGTGGTGGTAGCTGCTAC	………	TACTACTACTACTACGGTATGGACGTCTGG	20
	C A S	P	Y C S G G S C Y		Y Y Y Y Y G M D V W	
	**IgHV1-69**		D2-2*01		JH6*01	
**UCL6**	TGTGCGAGA	GGGATTCC	CCAGCCCCTA	CAGGAGGATA	ACTACTACTACGGTATGGACGTCTGG	19
	C A R	G I P	Q P L	Q E D	N Y Y Y G M D V W	

Sequence analysis of the IGHV1-69 homologous segment in six PCR clones isolated from the MAA-BSA panned umbilical cord blood Fab library. Determination of IGHV, IGHD and IGHJ usage and nucleotide sequence of HCDR3 in IGHV1-69.

### Analysis of HCDR3 Rearrangement of a Fab Clone from Phage Display Library

**Figure 4:**
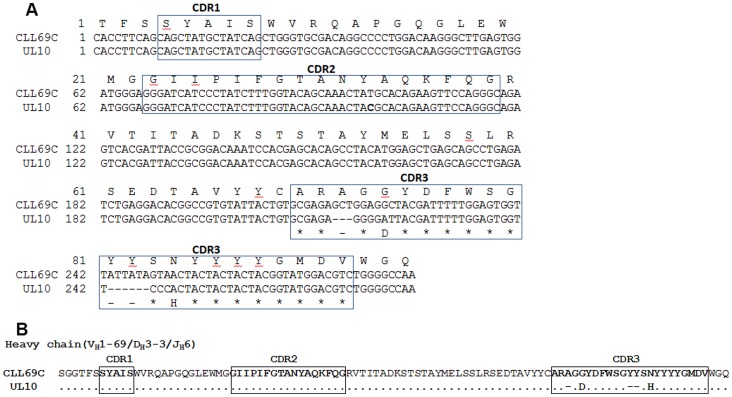
Sequence analysis and comparison of the IgHV of CLL69C to the highly homologous UL10 Fab. (**A**) Nucleotide and deduced amino acid sequence comparison of the UL10 Fab IgHV. Comparison was made with the closest CLL69C IgHV gene sequence. Dots indicate homology and dashes indicate no sequence at that position. (**B**) Amino acid sequence alignment of the IgHV of CLL69C with the UL10 Fab isolated from an umbilical-cord phage-display Fab library. Complementarity determining regions (CDR) are labeled above the amino acid sequence and indicated in bold.

The human umbilical cord Fab phage libraries pre-selected from 4th-round panning against MAA-BSA epitopes –both kappa and lambda- were further panned for binding to mAb G6 [25], [28], a murine anti-idiotypic mAb that recognizes unmutated IgH encoded by IGHV1-69. The nucleotide sequences of the IgH and IgL of one Fab clone that was isolated, designated UL10, were found to be nearly identical to the heavy chain of CLL69C (Fig. 4). The Ig heavy chain of UL10 is unmutated and encoded by IGHV1-69 (Fig. 4A), with a 20 amino acid long HCDR3 that is identical to UCL2, and is enriched in Tyr (Y), Gly (G), and Ser (S), prototypic of the HCDR3 of the unmutated CLL69C subset found expressed by CLL B cells. The HCDR3 includes nucleotide insertions both in the IGHV-IGHD junction (1 palindromic and 3 nucleotides) and IGHD-IGHJ junction (3 nucleotides) (Table 1). The IGHD3-3 gene and IGHJ6 gene are unmutated from the germline gene sequences. The finding of a CLL69C progeny in an OSE-selected Fab library isolated from newborns is consistent with the hypothesis that this Ig might be selected *in vivo* for binding to endogenous OSE.

### Expression of Active Fab Ab and Light Chain Shuffling Study

**Figure 5:**
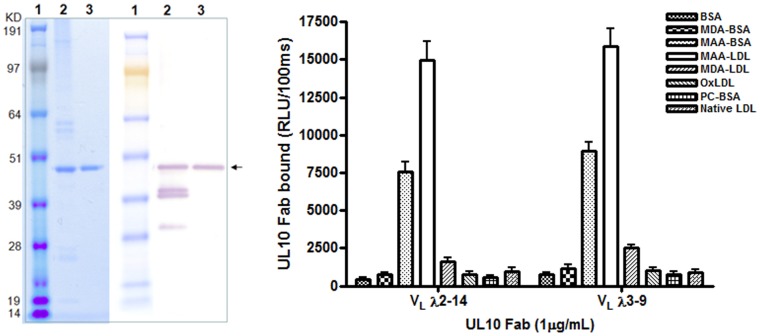
Expression and purification of active UL10 Fab Ab. (**A**) The UL10 Fab was produced in *E. coli* and purified by affinity chromatography from soluble fraction of *E. coli* lysate using nickel resin column. SDS-PAGE analysis of purified fractions under non-reducing condition and stained with Coomassie Blue (left three lanes), and blotted with anti-HA tag Ab (right three lanes). Lane 1, protein size standards (in kDa) shown at left; lane 2, *E. coli* extract expressing UL10 Fab; lane 3, purified UL10 Fab. (**B**) Binding pattern of CLL69C homologous UL10 Fab to OSE. Chemiluminescent ELISA for the binding of UL10 Fab (1 µg/ml) to each of the plated antigens (5 µg/mL) for 1.5 hour at RT. The data are expressed as RLU/100 ms and represent values from three independent experiments (mean ± SD), each value determined in triplicate.

The UL10 Fab clone was expressed in *E. coli* BL21 (DE3) cultures with 3′-terminal His_6_- and HA-double tags, and purified by affinity chromatography on a Ni^2+^-NTA-resin column and an additional step of anti-HA filtration chromatography. Immunoblots of SDS-PAGE of purified fractions under non-reducing conditions probed with anti-HA-tag Ab showed the near homogeneity of the Fab Ab fragment (Fig. 5, left panel). The purified UL10 Fab was soluble and functionally active, as it displayed the same pattern of binding reactivity to MAA epitopes as CLL69C rAb (data not shown).

Due to the nature of the combinatorial approach, light chain usage in combinatorial Fab clones was presumably random. The IgH and IgL chains of UL10 present in the Fab library may or may not have been originally paired in the human fetal IgM repertoire. To determine whether UL10 could form a functional anti-MAA Fab with alternative light chains, the UL10 IgL chain was exchanged with CLL69C light chain and evaluated for binding to MAA-LDL. Both the originally isolated UL10 (with IGLV2-14) and UL10 (paired with IGLV3-9 L chain of CLL69C) bound indistinguishably to the modified antigens, and specifically to MAA epitopes (Fig. 5, right panel). This demonstrates that Ig heavy chain of the CLL69C homolog Ab fragment (Fab) can also be paired with stereotypic CLL69C IgL and retain binding reactivity for the MAA-epitope.

### CLL69 Abs Bound to OSE on Apoptotic Cells

**Figure 6:**
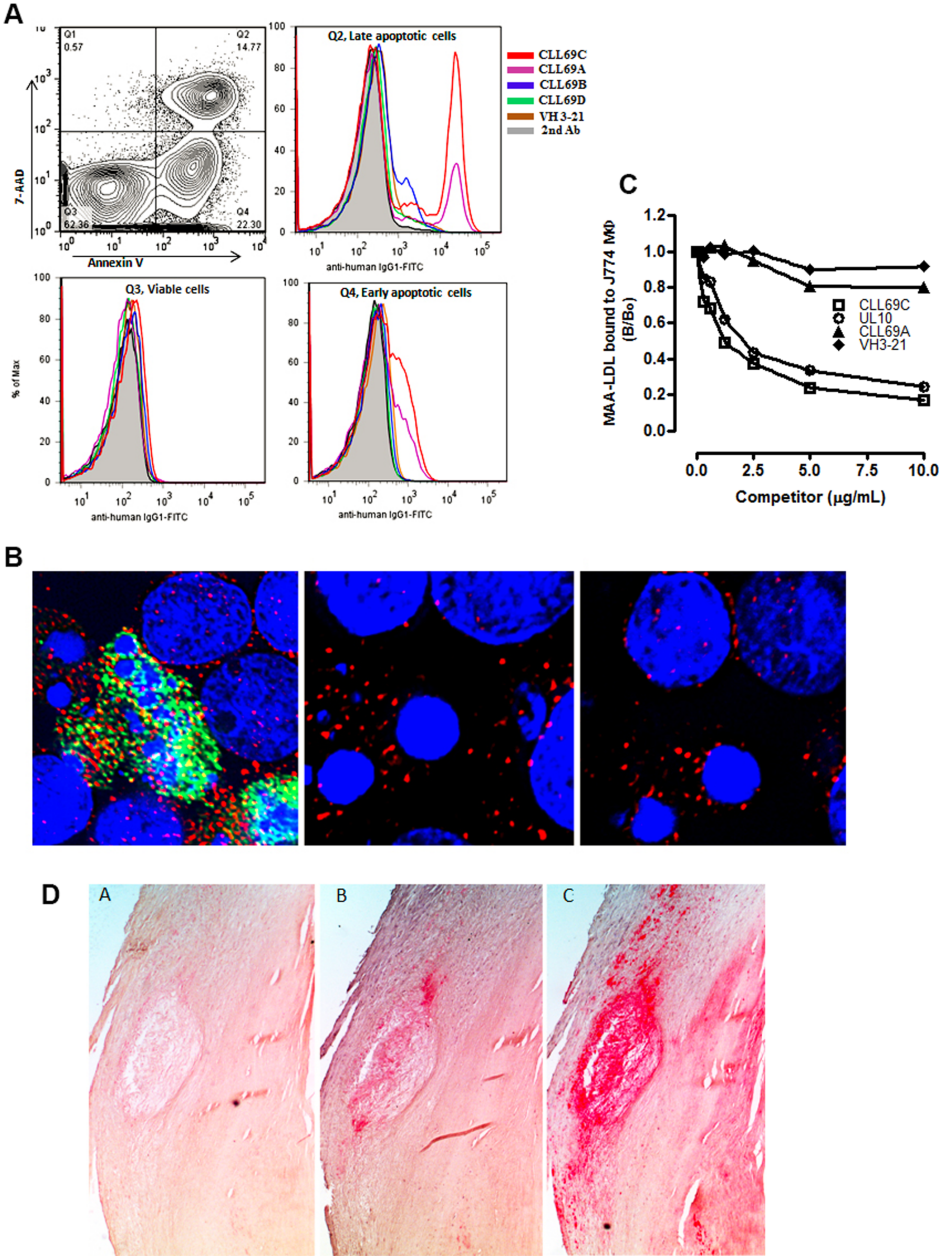
Binding of CLL69 Abs to OSE on apoptotic cells and atherosclerotic lesions. (**A**) Flow cytometry analysis of CLL69 rAb binding to apoptotic cells. Representative flow cytometry contour plots of apoptotic murine thymocytes and histogram plots for the binding of rAb to apoptotic cells. Murine thymocytes were induced to undergo apoptosis by incubation with 10 ng/ml PMA for 16 hours. Apoptotic cells were stained with rAbs CLL69 A–D, isotype VH3-21, or no primary Ab, followed by detection with a FITC-conjugated anti-human IgG1 and staining with PE-labeled Annexin-V and 7-AAD to identify the viable and apoptotic cells. The contour plot (upper left panel) identifies the viable cells (Q3, PE-Annexin-V^−/^7-AAD^−^), early apoptotic (Q4, Annexin-V^+^/7-AAD^−^) and late apoptotic cells (Q2, Annexin-V^+^/7-AAD^+^). Histogram panels Q3 (bottom left), Q4 (bottom right) and Q2 (upper right) represent rAb staining of viable cells, early apoptotic cells, and late apoptotic cells, respectively. Relative cell fluorescence of rAbs CLL69 A–D and IGHV3-21 control rAb are indicted by colored lines, and staining with the secondary Ab alone is shown in grey. (**B**) Immunofluorescence microscopy of CLL69C rAb binding to apoptotic cells. Deconvolution microscopy showing the binding of CLL69C rAb to late apoptotic Jurkat cells (FITC green color, the cell on the left panel) with dense fragmented nucleus detected by Hoechst staining (blue color), and apoptotic cells detected by PE-Annexin-V (red color), but not to a cell with intact nucleus (Panel A). The vector control and secondary Ab only (anti-IgG1-FITC) do not bind the apoptotic cells (panel B and C, respectively). (**C**) Inhibition of MAA-LDL binding to macrophage scavenger receptors by CLL69Ab. Competition assays for the inhibition of biotinylated-MAA-LDL binding to macrophage J774 by CLL69C rAb, UL10 Fab, or an irrelevant control VH3-21 rAb, as indicated. A fixed and limiting amount of biotin-MAA-LDL (5 µg/ml) was added to J774 macrophages in the absence or presence of increasing concentrations of indicated competitors. Extent of binding was determined in RLU/100 msec as described for chemiluminescent ELISA assays. Data shown represent the ratio MAA-LDL binding in the presence or absence of competitors (B/B_0_) as described in legend to Fig. 1B. Results are representative of three independent experiments, each point determined in triplicate. (**D**) Immunohistochemical staining of human carotid endarterectomy (CEA) specimens. Roughly parallel sections of human carotid endarterectomy tissue were stained with rAbs of CLL69C (panel C), CLL69A (panel B), or a secondary Ab alone (panel A). MAA-epitopes recognized by CLL69C or CLL69A are indicated by red color.

Oxidation-specific-epitopes are not only found in OxLDL, but are also prominent on apoptotic cells. We and others have shown the presence of a variety of OSE on apoptotic cells including the presence of oxidized phospholipids, and various MDA type epitopes including MDA and MAA [15], [29]. Indeed, all investigated murine and human NAbs display binding to apoptotic cells, particularly in late stages. Therefore, the binding of CLL69 rAb to apoptotic Jurkat cells and murine thymocytes was investigated using flow cytometry. Staining of Annexin V-PE and 7-AAD was used to separate apoptotic from non-apoptotic cells during flow cytometry. CLL69 rAb did not bind to viable cells with low Annexin V/7-AAD staining (Fig. 6A, Q3), but displayed increased binding to apoptotic thymocytes that had high Annexin V/7-AAD staining (late apoptotic cells, Fig. 6A, Q2). Figure 6A also shows that MAA-epitope specific CLL69C rAb bound more strongly to apoptotic thymocytes induced by PMA than other CLL69 rAbs. The specificity was verified by addition of 100 µg/mL MAA-BSA into the incubation medium, which almost totally abolished the CLL69C rAb binding to the apoptotic cells (data not shown). In addition, UV light exposure to induce apoptosis in Jurkat T-cells also resulted in similar binding of CLL69C (data not shown).

The flow cytometric analysis was confirmed by immunofluorescence microscopy, where CLL69C rAb bound to permeabilized late apoptotic cells (Fig. 6B, left panel). Using deconvolution microscopy, we demonstrated intense intracellular CLL69C rAb binding (FITC, green color) inside cells that had undergone apoptosis (fragmented dense nucleus visualized with blue Hoechst dye) and not to cells that had intact nucleus. No binding was observed with a control CLL rAb to the same cells (Fig. 6B, middle and right panel). These results support the hypothesis that OSE from dying cells, or their released apoptotic bodies or microparticles, could be commonly prevalent antigens leading to expansion of CLL69C B cells.

### Inhibition of Binding of MAA-LDL to Macrophages by CLL69C Abs

We previously demonstrated that many OSE-NAbs could bind to OxLDL and prevent binding and uptake of OxLDL by macrophages. Indeed, elevation of titers of certain OSE-Nabs, such as E06, was shown to be atheroprotective [30]. To evaluate if CLL69C rAb or UL10 Fab have functions similar to IgM NAbs, we examined the ability of these Abs to inhibit the binding of biotinylated MAA-LDL to macrophages. In prior studies, we showed that MAA-LDL binds specifically to macrophage scavenger receptors [15]. Biotinylated MAA-LDL was added to J774 macrophages in the absence or presence of increasing concentrations of CLL69C rAb, UL10 Fab, or control Ab, and the extent of binding was determined (Fig. 6C). Both CLL69C rAb and UL10 Fab competed for MAA-LDL binding in a dose dependent manner, while neither CLL69A or control IGHVH3-21-encoded rAb bound, demonstrating the specificity of CLL69C -derived rAbs to the MAA-LDL adducts (Fig. 6C).

### CLL69 Abs Recognized OSE in Atherosclerotic Lesions

As we and others [18] have shown that MAA epitopes are enriched in atherosclerotic lesions, we examined if CLL69C rAb could immunostain such lesions. CLL69C rAb strongly immunostained atherosclerotic lesions from human carotid endarterectomy (CEA) specimens (Fig. 6D) or WHHL rabbit aortas (Figure S2). In particular, the necrotic cores of the lesions were strongly stained with CLL69C rAb, but not control Ab (Figure S2), similar to observations obtained in our lab with the MAA-specific NAb LRO4 (data not shown).

## Discussion

In this study we analyzed the binding reactivity of several CLL derived sterotypic IGHV1-69-encoded rAb to multiple oxidation specific epitopes, which are a major target of innate NAbs. We found that IGHV1-69-encoded rAb, particularly CLL69C or CLL69A, bound to OSE, and more specifically to the immunodominant OSE adduct MAA, which is found on apoptotic cells, inflammatory tissues, or atherosclerotic lesions. CLL69C rAb also reacted specifically with MAA-specific peptide mimotopes. Light chain shuffling indicated that non-stochastically paired CLL69C IgL encoded by IGLV3-9 contributes to the antigen binding of CLL69C. Furthermore, nearly identical CLL69C IgH were isolated from an MAA-enriched umbilical cord IgM phage displayed Fab library, indicating the presence of these conserved IgH in the early, developing B cell repertoire.

Molecular mimicry between microbial pathogens and neo-self Ag has been suggested to be a critical contributor to the activation of autoreactive B cells [31], [32]. The IGHV1-69 gene has also been shown to be preferentially used by Ig heavy chains of the Ab generated in a number of viral and bacterial infections and autoimmune disorders, such as neutralizing antibodies to the HIV gp120 glycoprotein [33], and hemagglutinin of influenza subtypes [34], as well as in anti-DNA and cardiolipin autoantibodies from patients with systemic lupus erythematosus [22], [35]. In concert with other immunomodulatory mechanisms, such as B cell activation and recurrent stimulation with autoantigens that display molecular mimicry with exogenous pathogens [36], [37], these immune responses may escape regulation, leading to the formation of pathogenic autoantibodies, suggesting that common antigenic stimulation could cause the unconstrained expansion of activated IGHV1-69-expressing B cells.

The rAbs that belong to the CLL69A subgroup (IGHV1-69/IGKV3-20) are virtually identical to a natural Ab of IgM IGKV3-20 class with anti-cardiolipin/rheumatoid factor (RF factor) activity and low-affinity for a variety of self-antigens (RF activity and bind to myoglobulin, actin, and ssDNA) [38]. IGHV1-69/IGKV3-20 expressing Ig are also over-represented among natural IgM Abs that cause mixed cryoglobulinemia associated with hepatitis C virus infection [39]. The CLL69C Ab resembles the BCR from a group of CLL cases (CLL22, CLL17, and CLL72) using the HCDR3 rearrangement (IGHV1-69/IGHD3-3/IGHJ6) and IGLV3-9-encoded light chain reported previously [40]. Among 21 cases that express the CLL69C HCDR3 motif, four were identical and 17 others had marked CDR3 homology in both IgH and IgL chains, which might reflect selection for specific structural motifs that facilitate antigen binding. The remarkable similarity for the known hypervariable HCDR3 regions within multiple CLL patients strongly supports the view that particular antigen reactivity is the selective force. The expression of such conserved BCRs with restricted HCDR3 in CLL disease suggests a potential role for antigen stimulation in the development and/or progression of CLL.

Oxidation-specific epitopes represent prominent examples of oxidative, stress-induced altered self. These OSE are generated ubiquitously as a consequence of lipid peroxidation during many physiological and pathological situations, including leprosy, diabetic nephropathy, hepatosteatosis, various CNS diseases (including multiple sclerosis and Alzheimer), rheumatoid arthritis, and carotid, femoral, or coronary atherosclerosis [14]. OSE are also prominently generated in inflammatory viral and bacterial diseases, and may share molecular mimicry with epitopes on pathogens [14]. Importantly, trillions of apoptotic cells are generated daily and OSE are prominently found on the membranes of apoptotic cells, shed apoptotic bodies and microparticles.

Among the many OSE generated, oxidized phosphocholine (PC) phospholipids that display the PC epitope, which shares molecular identity with the PC of pathogens, is a common target of many NAbs, such as E06, which we have extensively characterized, as well as CLL BCRs [35], [36]. Malondialdehyde is an even more ubiquitous end product of lipid peroxidation and readily forms both simple and complex adducts with proteins and other lipids. Among these, we have shown that as a class, such OSE are a major target of innate NAbs. Because many CLL BCRs display gene usage consistent with NAbs, it is not surprising then that many such BCRs bind to OSE. Indeed, several recent reports document that OSE, such as the PC of OxLDL and bacteria, and MDA, are common target antigens to which the BCRs of many CLL might bind [35], [36].

Among different MDA-type adducts, we and others have shown that MAA epitopes are immunodominant, being detected in a variety of inflammatory tissues, including alcoholic hepatitis, atherosclerotic aortic lesions, and apoptotic cells [15], [16]. Remarkably, we have recently shown that MAA epitopes are the target of ∼ 15% of all IgM NAbs in mice and in human newborn cord-blood [15]. Here we demonstrate that CLL69C specifically recognizes MAA epitopes, as shown by direct binding assays (Fig. 1A) as well as in competition immunoassays (Fig. 1B). Furthermore, it also had the biological property of inhibiting the binding of MAA-LDL to macrophages (Fig. 6C). CLL69C also specifically immunostained apoptotic cells (Fig. 6B) and both human and rabbit atherosclerotic tissue (Fig. 6D, Fig. S2). Thus, it appears to have all of the immunological binding properties of numerous OSE NAbs we have studied. These observations suggest the possibility that the continual generation of these ubiquitous epitopes throughout life contributed to the positive selection of CLL B-cells that express Ig with such specificities. Furthermore, light-chain shuffling tests (Fig. 2) indicated that both the CLL69C Ig heavy and Ig light chains contributed to the binding activity of rAb for MAA epitopes. Indeed, in a recent report, Catera *et al.* [36] also noted that CLL BCRs with heavy chains that had similar HCDR3 gene usage (IGHV1-69/IGHD3-03/IGHJ6) but with different light chains, also bound to apoptotic cells, and had weak binding to MDA-BSA, but they did not test binding to MAA.

In support of the hypothesis that CLL69C was selected by ongoing MAA-epitope exposure, we demonstrated the presence in a human fetal Fab library of unmutated ancestor IGHV1-69 germline domains (CLL69C). We utilized PCR and anti-idiotypic mAb to IGHV1-69 to investigate the ontology of CLL69C gene lineage maturation and clonal expansion from the fetal BCR repertoire. The UCL2, UCL3, and UL10 clones were isolated from an MAA-enriched phage library, and were found encoded by unmutated IGHV1-69 germline genes (CLL69). We produced a Fab clone with the UL10 IgH chain paired with the CLL69C light chain to generate a soluble Fab Ab and found that this Fab also bound prominently to MAA epitopes (Fig. 5). Further evidence for the existence of CLL69C-like heavy chains of normal B cells is provided in a study that analyzed the nucleotide sequences and primary molecular structure of 143 IGHV1-69-encoded IgH that were derived from the B cells of three healthy adult donors [13]. They found that 22 (15%) of the IgH could be classified into a previously identified HCDR3 subgroup, including four that were assigned to the CLL69C analogous subgroup 7. No IgH belonging to the CLL69-A subgroup were identified, as the analysis was specific for identifying IgH with longer than average HCDR3 - encoded by IGHJ6. In aggregate, these data further advance the hypothesis that host MAA-epitopes could drive clonal expansion of stereotypic subsets of CLL69C B cells with optimal binding for MAA. Presumably these continually proliferating B cells subsequently escape vital checkpoint regulation, resulting in the unregulated clonal expansion leading to CLL [41], [42], [43].

As noted above, MAA-epitopes have been identified as immunodominant epitopes of MDA-modifications, and that there are high-titered MAA-specific IgG and IgM Abs in human plasma. We recently identified small peptide mimotopes that mimic MAA-epitopes in MAA-LDL, which in turn were recognized by LR04, an MAA-specific monoclonal Ab [20]. Similar to LRO4, we showed that CLL69C bound to these MAA-mimotopes, and in particular to the P1 mimotope in a dose-dependent manner (Fig. 3B), and that this binding was efficiently inhibited by increasing concentrations of soluble MAA-BSA (Fig. 3C). Thus, the MAA mimotope P1 might have potential in diagnostic applications to identify MAA-specific Abs, such as CLL69C. The availability and reproducibility of these small peptide mimotopes could facilitate the development of standardized clinical assays, allowing the rapid identification of such BCRs on CLL B cells. The CLL69C gene analyses and MAA- binding levels may be an important prognostic indicator, along with other biologic markers, for prediction of CLL disease outcome.

## Supporting Information

Figure S1 CLL69C rAb exhibited dose-dependent binding to MAA-epitopes. CLL69A and CLL69C rAbs both exhibited dose-dependent binding to plated MAA-LDL (left panel A) and MDA-LDL (right panel). Shown are the extent of binding (in RLU/100 msec) of the indicated concentration of Abs. Values shown are the mean ± SD of triplicate determinations of three independent experiments.
(TIF)

Figure S2 Immunohistochemical staining of atherosclerotic lesions with CLL69C rAb. Atherosclerotic lesion obtained from the aorta of a hypercholesterolemic rabbit immunostained with CLL69C rAb (Panel A) or with a secondary Ab control (Panel B). Epitopes recognized by CLL69C are indicated by red color and nuclei are counterstained with hematoxylin. These data are similar to immunostaining with LRO4, a known anti-MAA NAb cloned in our lab (data not shown).
(TIF)
