# Supplementary figures and images for: IGHV1-69-Encoded Antibodies Expressed in Chronic Lymphocytic Leukemia React with Malondialdehyde–Acetaldehyde Adduct, an Immunodominant Oxidation-Specific Epitope
Source: PLoS One. 2013 Jun 20;8(6):e65203. doi: 10.1371/journal.pone.0065203 (PMC3688726; doi:10.1371/journal.pone.0065203)

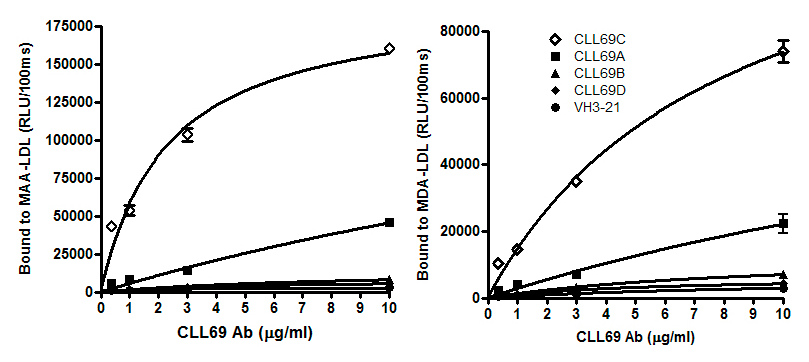

Supplement: Figure S1 — CLL69C rAb exhibited dose-dependent binding to MAA-epitopes. CLL69A and CLL69C rAbs both exhibited dose-dependent binding to plated MAA-LDL (left panel A) and MDA-LDL (right panel). Shown are the extent of binding (in RLU/100 msec) of the indicated concentration of Abs. Values shown are the mean ± SD of triplicate determinations of three independent experiments. (TIF) [file pone.0065203.s001.tif]

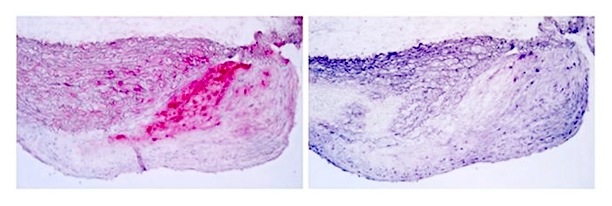

Supplement: Figure S2 — Immunohistochemical staining of atherosclerotic lesions with CLL69C rAb. Atherosclerotic lesion obtained from the aorta of a hypercholesterolemic rabbit immunostained with CLL69C rAb (Panel A) or with a secondary Ab control (Panel B). Epitopes recognized by CLL69C are indicated by red color and nuclei are counterstained with hematoxylin. These data are similar to immunostaining with LRO4, a known anti-MAA NAb cloned in our lab (data not shown). (TIF) [file pone.0065203.s002.tif]
